# Supplementary material for: Entropic Origin of Pseudogap Physics and a Mott-Slater Transition in Cuprates
Source: Sci Rep. 2017 Mar 22;7:44008. doi: 10.1038/srep44008 (PMC5361159; doi:10.1038/srep44008)
Supplement: Supplementary Information [file srep44008-s1.pdf]

SUPPLEMENTARY MATERIAL for
Entropic Origin of Pseudogap Physics and a Mott-Slater Transition in Cuprates

R.S. Markiewicz, I.G. Buda, P. Mistark, C. Lane, and A. Bansil

PACS numbers:

SI. ORDER PARAMETER CLASSIFICATION SCHEME

A. Classifying order parameters

Our order parameter classification scheme is based on the existence of a generalized Stoner criterion for phase instability,

$$V_q\chi_0(q,\omega=0)=1+\lambda. \tag{S1}$$

The restriction to zero frequency means that we cannot discuss quantum critical phenomena; we shall see that the formulation works best at higher Ts. In analyzing Eq. S1, we assume that the q dependence of V_q is weak. This is certainly true in the Hubbard model. For the bare Coulomb interaction V_q behaves like a power law, e.g., $V_q \sim 4\pi e^2/q^2$ in 3D, and the only special q values are 0 and ∞. We can then calculate *fluctuation maps* that divide the $T-x$ or the $\omega-x$ phase space into *basins of attraction*, where the q-vector corresponding to the maximum $\chi_0(q,0)$ value changes character. Examples of fluctuation maps are shown in Figs. 3 and 4 of the Main text. Phase transitions have a different character depending on whether they arise within a basin or are due to transitions across the boundary between different domains. In spectrum generating algebras, this boundary is referred to as a *separatrix*.[1,2]

For phase transitions within a single basin, the transition is controlled by the net electron degeneracy, and hence typically by an SO(N) point group, and by the corresponding Us that break the degeneracy. For example, if we consider density wave fluctuations at a particular Q, the 1 on the right hand side of Eq. S1 is appropriate for a SDW, but shoud be -1 for a CDW. The difference is an issue of charge neutrality. The Coulomb energy is so large that if electrons bunch up into a CDW it will cost an energy comparable to the plasma energy, unless V is attractive. On the other hand, in an SDW the up and down spins bunch up out of phase, so the magnetization oscillates in space while the charge density is constant, and hence condensation is easier. The same effect holds if there is an orbital or valley degeneracy. Moreover, in a Peierls-like scenario, the electron bunching is accompanied by a lattice distortion controlled by a Stoner condition equivalent to Eq. S1, with an effective interaction U_{eff}. For the cuprates, the two VHSs at $(\pi,0)$ and $(0,\pi)$ act as a form of valley degeneracy, and the theory with $q=(\pi,\pi)$ is controlled by an SO(8) group. Similarly, for antinodal nesting the (δ,δ) density wave can be either SDW or CDW.

A new result of our investigation is the possibility of *supertransitions*, where doping or temperature causes the system to cross a separatrix between different basins of attraction. In the theory of spectrum generating algebras these are known as *finite energy quantum critical points*, and it is found that an *order-parameter DOS* diverges at the transition. We find that we can reproduce similar behavior by defining the order-parameter DOS as the SDOS at threshold, which controls the singular behavior of ξ [Results Section (Beyond RVB) of the Main text].

Finally, we discuss how the fluctuation maps can be used to generate phase diagrams. At mean field level, a phase transition occurs when the generalized Stoner criterion of Eq. S1 is satisfied, so that if V_q is a constant, the transition would fall along one of the contour lines (black dots) in Fig. 2 of the Main text, and even if V_q varies with x and/or T, these lines can be used to find the transition. For a 3D system, this will simply mean that mode coupling depresses the transition temperature to a lower T where the Stoner criterion is satisfied, leaving behind an extended pseudogap regime. Once the system develops long-range order, the Fermi surface and susceptibility change, and the fluctuation map at lower T must be recalculated. Does this mean that transitions predicted from the fluctuation maps are inaccessible? If the lower-T phase actually has the lower free energy, a transition might occur spontaneously, or the system might get trapped in a metastable state. In this case, there are two possible ways of observing these states. First, since there is an equivalence class of materials with the same fluctuation map, it is possible that different members of the class have smaller values of V_q, for which the Stoner criterion is only satisfied in the lower-T phase. Alternatively, one might try to quench or photoexcite the system.

For a 2D system, the issue is more subtle as the Mermin-Wagner theorem holds and an ordered phase can only arise at $T=0$ unless the system transitions to 3D or its spin dimension decreases due to spin-orbit coupling. Thus, when the correlations of one phase start to grow, one will need to account for the evolution of the Fermi surface and susceptibility at lower T. This can be complicated as each competing density wave corresponds to a different Fermi surface reconstruction. Here we note one point in particular. In the Main text, Fig. 15(c), we see the development of an ANN phase out of a short-range order $(\pi,\pi-\delta)$ SDW in good agreement with the CDW phase found in many cuprates. Since the SDW correlation length is only $\xi_{SDW}\sim 5a$, this is perhaps not too surprising, but for lower x ξ_{SDW} should grow, and the developing small Fermi surface should be taken into

account. One issue is whether the CDW develops homogeneously or on the SDW domain walls. In this connection we note some experimental evidence of a possible relevant phase found in another cuprate. In LSCO, the low-temperature tetragonal (LTT) phase is believed to be highly relevant to stripe physics, anchoring the direction of the stripe to the lattice and producing the unusual criss-cross stripe pattern along the c-axis. Yet the LTT phase is almost never the stable phase, existing as fluctuations or as a minority phase, although it can be stabilized via rare-earth substitution. Electron microscopy studies find that the LTT phase tends to form as inclusions or on twin-boundaries of the low-temperature orthorhombic phase[3,4], suggesting that quenching these materials into the low-T phase might prove interesting.

Lastly, one can apply tuning parameters to selectively turn an order on or off. We have seen that disorder can be used to quench the ANN phase. Moreover, magnetic fields are regularly used to turn off superconductivity to reveal the competing phases. A magnetic field has an inordinately strong effect in destroying spin-singlet Cooper pairs. For instance, at high fields, superconductivity is often restricted by the Clogston-Chandrasekhar limit[5,6], $\mu_B H = \Delta(0)/\sqrt(2) \sim 1.76 k_B T_c/\sqrt(2) \sim 2.6$meV, assuming $T_c = 30$K, superconducting gap $\Delta(T)$, and the BCS ratio for $2\Delta(T=0)/k_B T_c$. Since the Bohr magneton is $\mu_B = 0.057$meV/T, this gives a critical field 46T, in good agreement with experiment in LSCO[7]. In contrast, magnetic fields have relatively weak effects on antiferromagnetism[8]. Typically, one expects a spin-flop transition at fairly low fields, where the AF moment aligns perpendicular to H, followed by a gradual rotation of the moments until they are parallel to B and the AF order is destroyed. While a weak spin-flop transition is often observed in the undoped cuprates, associated with Dzyaloshinskii-Moriya induced canting of the moments, the main effect of the field is only expected near the saturation field, $\mu_B H \sim J \sim 100$meV ~1800T.

B. $x - \omega$-scaling

It is important to note that for a Fermi liquid, the scaling in the $x-T$ space found in the Main text (Fig. 9) implies a similar scaling in the $x-\omega$ space. In fermionic systems there is a similar scaling in T and ω, with scale-factor

$$\hbar\omega \sim a k_B T, \qquad \text{(S2)}$$

$a \sim \pi$. Here we compare the $x-T$ map of Fig. M9 with the corresponding $x-\omega$ maps, which can be more readily compared with the SGA results. First, in Figs. S1 and S2 we compare plots of susceptibility curves varying x at either $T=0$ and fixed ω (Fig. S1), or at $\omega = 0$ for several values of T (Fig. S2). The latter were used to calculate the light-blue dot-dashed line in the Main text, Fig. 9. For each ω, starting at electron doping ($x < 0$), there is a peak at (π, π) which grows with x, reaches a maximum, then starts to broaden and decrease, ultimately splitting into two peaks with the stronger peak at $(\pi, \pi - \delta)$. Figure S3 plots the evolution of the peak (red solid line) and the onset of the split bands (blue line). The blue squares are the data of Fig. 9 of the Main text, scaled by a factor $a = 5$, showing that Eq. S3 is approximately satisfied. The thin red lines represent constant-χ contours, labeled by the Stoner U value. Once again, as U decreases, the unstable region converges onto the VHS doping. We note that the onset of splitting (blue line in Fig. S3) is somewhat uncertain, as the peaks are quite broad and it is unclear just where they start to split.

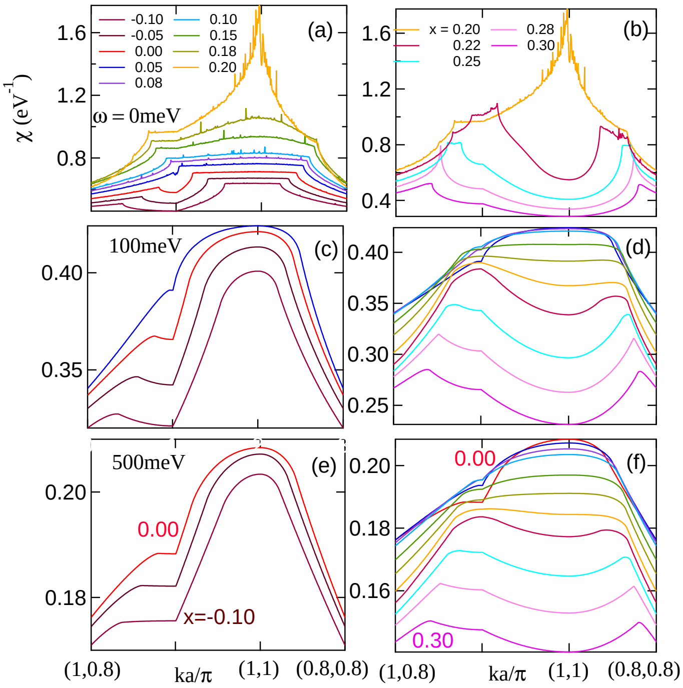

FIG. S1: **Doping dependence of χ_0 at fixed ω and $T = 0$.** Left hand frames are for $x < x_{peak}$ and right hand frames for $x > x_{peak}$. Value of x is color coded according to legends in frames (a) and (b), and x_{peak} is plotted in Fig. S3. Frequency $\omega = 0$ (a,b), 100 meV (c,d), and 500 meV (e,f). Calculations are based on full LDA-based dispersion for LSCO.

C. Relation to spectrum-generating algebras

Spectrum-generating algebras (SGAs)[1,2] were developed in the context of nuclear physics to elucidate the underlying group structure of order parameters, taken as bilinears of creation and annihilation operators. The primary concern of SGAs is not with interactions, but with the underlying topology of the order parameters, defined by the group structure of bilinears. The Lie algebra contains chains of subalgebras, which define do-

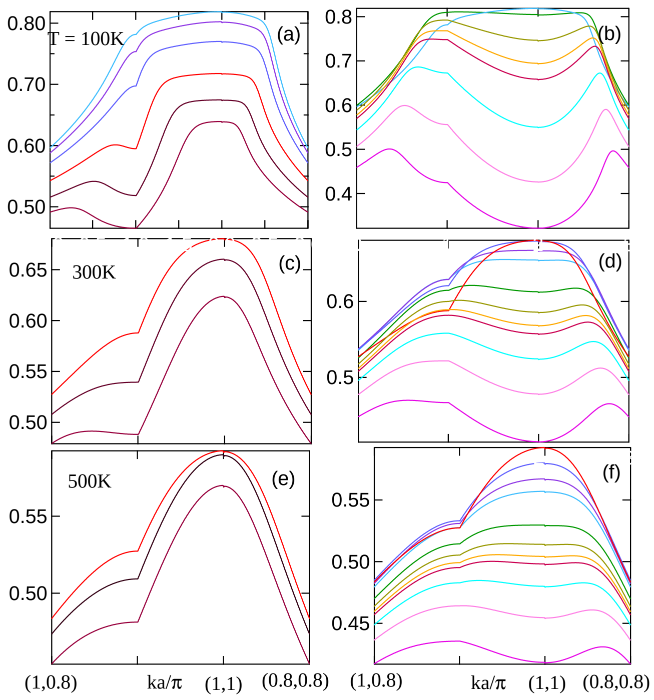

FIG. S2: **Doping dependence of χ_0 for fixed T at $\omega = 0$.** Setup and doping color scheme is the same as for Fig. S1, except temperature T = 100K (a,b), 300K (c,d), and 500K (e,f).

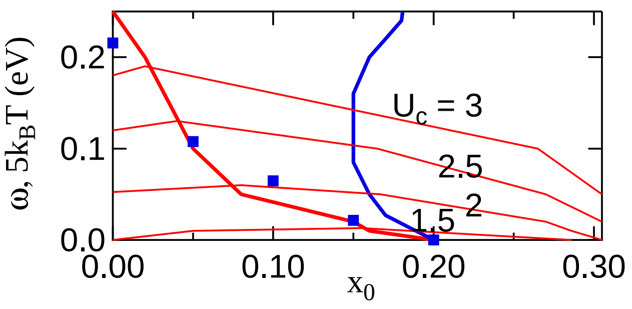

FIG. S3: **Commensurate-incommensurate crossover in the $x-\omega$ space.** Plot of x_{peak} as a function of ω, taken from Fig. S1 and related calculations (red solid line), compared to the scaled results from the Main text, Fig. 9 [blue squares], and of the commensurate-incommensurate transition (blue solid line). Thin red lines indicate contours of constant χ_0, labeled by their equivalent values of $U_c = 1/\chi_0$. All curves are plotted vs frequency ω, except the blue squares, which are plotted vs $5k_BT$.

mains of attraction for the order parameters. Within each subalgebra, more conventional QCPs are driven by symmetry-breaking interactions which couple to individual elements of the subalgebra. SGAs are less concerned with the ordinary quantum critical points (QCPs) than with 'supertransitions' across the separatrix between domains, brought about by external perturbations. At this crossover, states of both subalgebras are degenerate, leading to divergent 'order-parameter-DOSs' and possible emergent behavior. While phase transitions occur in SGAs at zero frequency, the domains and separatrices persist at all energies, leading to the concept of excited-state quantum critical points (ESQCPs). One introduces a perturbation ξ that switches the ground state from one subgroup to another, then plots the eigenvalue spectrum from one sector of the ground state (say, the states with angular momentum quantum number $l = 0$) as ξ is varied. One thus finds a separatrix line in the $E-\xi$-space where the eigenvalues pile up, leading to a peak in the corresponding density-of-states. As the eigenvalues cross this line, degenerate eigenvalues on one side split on the other side, signaling a change in the ground state. As $E \to 0$, the separatrix extrapolates to the conventional QCP, but at finite E it becomes an ESQCP.

Attempts to extend the ideas of SGAs into condensed phases have run into difficulties. A key difference between the SGAs in nuclear and condensed matter physics is that the former are designed with nearly-spherical nuclei in mind, and the associated groups are generally related to SO(N) and SU(N), whereas in solids, the pairs are first sorted by a q-vector, which is then superposed with the group of the star of each q.

In Section SI.A we showed that the DFT-Lindhard susceptibility can be used to extend these ideas into the condensed matter domain. Since the susceptibility χ_q can be identified with the order parameter $\sum_k < c^\dagger_{k+q} c_k >$, the fluctuation fingerprints bear a close resemblance to the domains of attraction in SGAs, with doping x acting as a symmetry-changing parameter similar to ξ. In this case, it is natural to identify the threshold value of the SDOS [corresponding to the largest susceptibility or the smallest inverse susceptibility] as the equivalent of the order-parameter DOS of SGA. The Main text Figs. 14(c-e) show that the threshold SDOS diverges at the Mott-Slater crossover. This divergence persists at finite T, suggestive of an ESQPT. The emergent behavior we find at the transition is thus a form of deconfined QCPs (DQCPs), with a non-Landau-Ginzburg-Wilson transition between two types of competing order, where a new form of excitation emerges exactly at the DQCP.[9,10] We show in the Main Results Section (Beyond RVB) that the SDOS also diverges at the separatrix between (π,π)-order and the 'ring' phase.

If we interpret the pseudogap as condensation into the coherent (π,π)-domain, then the low-temperature physics will be controlled by the SO(8) algebra of the VHS.[11] This algebra contains two SO(6) subalgebras of high interest to cuprate physics, one that combines

Zhang's SO(5)[12] [SDW plus d-wave superconductivity] with a flux phase operator, and a second with CDWs, spin-flux phase, and s-wave superconductors. These two SO(6) vectors are coupled by a nematicity operator. We see that this Lie algebra contains all of the low-T phases found in the cuprate pseudogap.

In summary, we have constructed the condensed-matter analog of the spectrum-generating algebra, and shown that the characteristic frequency dependencies are echoed in the T-dependence, and hence experimentally accessible. In particular, pseudogap physics in LSCO is dominated by the VHS domain of attraction, and the low-T ordered phases are associated with the corresponding $SO(8)$-algebra.

SII. INTERPRETATION OF SELF-CONSISTENCY

A. Relation to Entropy

In Landau theory, fluctuations above the ground state are typically calculated in Gaussian approximation[13], yielding an entropy in the paramagnetic phase

$$S = S_0(E) - \frac{1}{2T}\sum_{q,i}\chi_q^{-1}|\phi_{q,i}|^2, \tag{S3}$$

yielding a free energy

$$F = F_0 + \frac{1}{2}\sum_{q,i}\chi_q^{-1}|\phi_{q,i}|^2, \tag{S4}$$

where $\phi_{q,i}$ $(i = x, y, z)$ is the amplitude of the magnetic fluctuation. Then if each degree of freedom of F has an average energy of $T/2$, then $< |\phi_{q,i}|^2 >= T\chi_q$, so the total fluctuation intensity is

$$\sum_{q,i} < |\phi_{q,i}|^2 >= 3T\sum_q \chi_q \propto \lambda. \tag{S5}$$

This clearly demonstrates that our calculation is a direct generalization of McMillan's phononic entropy[14] to the situation of electronic bosons.

B. Relation to Bose-Einstein Condensation

In the conventional picture of Bose-Einstein condensation (BEC), the total number of bosonic modes must satisfy

$$N = \int d\omega n_{BE}(\omega) D_B(\omega), \tag{S6}$$

where n_{BE} is the BE distribution function and D_B is the bosonic density of states. As T decreases, the bosonic chemical potential μ must adjust to maintain the equality. However, the largest μ can be is zero, for which $n_{BE} \rightarrow n_P$, the Planck distribution, and the RHS of Eq. S6 takes on a fixed value. For lower T, the equation can only be satisfied by putting a macroscopic number of bosons into the lowest energy mode.

In the present situation, $D_B = \sum_q \chi''$, and n_P can be approximated as $\sim T/\omega$, in which case Eq. S6 becomes

$$N \simeq T\sum_q \int d\omega \frac{\chi''(q,\omega)}{\omega} = \pi T\sum_q \chi'(q,\omega=0). \tag{S7}$$

Comparing this to Eq. 5 in the Main text, self-consistency has produced a number $N_{eff} = N\pi\lambda/A_0$ of 'excitons' which can subsequently Bose condense. Whereas for 3D BEC the macroscopic occupation of the lowest bosonic mode must be added by hand, in 2D the self-consistent condition automatically causes the occupation of that mode to diverge as $T \rightarrow 0$.

C. Entropy and Dissipation

As originally noted by McMillan[14], anomalously large ratios of $2\Delta/k_BT_c$ for any phase transition are generically associated with a dominance of bosonic entropy at finite T due to low-lying bosonic fluctuations. But, from the fluctuation-dissipation theorem, the net fluctuations at a given frequency ω are equal to the dissipation $D(\omega)$ at that frequency,

$$D(\omega) = \sum_q \chi''(q,\omega)(n_{BE}(\omega) + 1/2) \rightarrow T\sum_q \frac{\chi''(q,\omega)}{\omega}, \tag{S8}$$

so that the right-hand side of Eq. S7 is the total dissipation at temperature T. In an ordered phase near $T = 0$, the dominant fluctuations will be associated with Goldstone modes, but to show this in a calculation requires extending the present results to full self-consistency between the self-energy and vertex corrections as in a parquet calculation.

SIII. SAMPLE MODE COUPLING CALCULATION

Simple numerical integration of the Main text Eq. 14 cannot easily handle the logarithmic singularity near $X_- = U_c$. Hence, we rewrite $N_- = N_1 + N_2$, where N_1 has a simple analytical form and captures N_- near the threshold, $N_2 << N_1$ for small $(X_- - U_c)$. Then the integral $I = \int N_- dX_-/(\delta + (X_- - U_c))$ is split into $I_1 + I_2$, corresponding to the two N_i, and I_1 is evaluated analytically, while I_2 (which is nonsingular) is evaluated numerically. For example, at higher T, N_1 varies approximately linearly with X_- near the threshold, $N_1 = N_a - N_b(X_- - U_c)$ for $0 < (X_- - U_c) < X_c$.

In this case,

$$I_1 = (N_a - N_b\delta)ln(\frac{X_c}{\delta}+1) + N_bX_c. \quad \text{(S9)}$$

For small δ this reduces to the OZ form $I_1 \sim N_aln(\frac{X_c}{\delta})$, thereby describing Region I of the Main text Fig. 13(b).

For the small-t' materials, the susceptibility on the plateau remains parabolic, but with a small curvature and a sharp cutoff. This leads to an additional contribution to the SDOS of the form $N_- = N_p$ if $(X_- - U_c) \leq X_p$. Then the integral of Eq. S9 contains an extra contribution

$$I_p = N_pln(\frac{X_p}{\delta}+1). \quad \text{(S10)}$$

This term has two distinct limits. At low T, $\delta << X_p$, in which case $I_p = N_pln(X_p/\delta)$. Combining this with I_1 leads to an OZ-like result of the same form but with a larger step height, $N_a \rightarrow N_a + N_p$, describing Region III of the Main text Fig. 13(b). However, as T increases, δ grows rapidly, leading to a possible reversal of the inequality. If $X_p << \delta$, then $I_p \rightarrow N_pX_p/\delta$. This is equivalent to assuming that the plateau has a flat top, in which case N_- can be represented as a δ-function, $N_- = N_pX_p\delta(X_- - U_c)$, with N_pX_p the excess height of N at threshold, which is proportional to the area of the plateau at $T = 0$. From the Main text Eq. 14, $I_p \sim 1/\delta$ translates into $\xi_{th} \sim 1/T^{1/2}$, dotted line in the Main text Fig. 13(b), which approximately describes region II.

SIV. RELATION TO OTHER CALCULATIONS

A comparison between our QPGW model and other 'DFT+' calculations was given in Ref. 15; here we discuss this comparison specifically in regard to pseudogap physics. A number of groups are working on MBPT-type extensions of DFT calculations. However, DMFT and its cluster extensions are limited by a very low resolution in momentum q. In particular, this means that even if one captures Mermin-Wagner physics, one will be typically limited to $\xi < 7a$[16] [Fig. 50 of Ref. 15]. Furthermore, in averaging over large patches in momentum space, one loses the ability to resolve ordinary nesting instabilities, such as the ANN instability responsible for the cuprate CDWs [Fig. 49 of Ref. 15]. On the other hand, the fact that one can see the pseudogap, even when averaging over a quarter of the Brillouin zone is consistent with our finding that the pseudogap is spread over many separate modes in q. Notably, whereas we find that the pseudogap is related to a peak in γ, recent cluster-DMFT calculations find that the pseudogap transition is associated with a line of peaks in the compressibility (Ref. 17 and references therein). Since the Fermi liquid compressibility is proportional to the DOS, this line would also be expected to terminate at a VHS, consistent with our results. However, since the cluster-DMFT calculation assumed $t' = 0$, the (paramagnetic) VHS would be at $x = 0$, whereas the $T = 0$ compressibility peak lies at a finite doping. We suspect that it is related to the VHS of the bonding AF band. The cluster-DMFT calculation reveals an additional complication: within the superconducting state the compressibility diverges, leading to a regime of phase separation (see also Ref. 18).

Some DMFT extensions [dynamic vertex approximation[19–21], dual fermion[22,23], and one-particle irreducible approaches[24]] seek to calculate the two-particle vertex, and hence the momentum-dependent self energy, and are able to reproduce Mermin-Wagner physics and vertex corrections. In particular, a recent dynamical cluster approximation (DCA)[25] calculation sorted out the contribution of various channels of two-particle scattering to the self-energy ('fluctuation diagnostics'), and found that spin fluctuations are the origin of the pseudogap, while charge and pairing fluctuations play a marginal role, consistent with our results. This has also been found experimentally[26].

We further note that the parquet equations[27,28] could be quite useful, if the recently discovered divergences[25] can be overcome. Mutual self-consistency between the self-energy and vertex corrections will be necessary to extend the current results to low temperatures.

After our paper was posted [arXiv:1505.04770], we became aware of several related calculations. In particular, Ref. 29 confirms the existence of an electronic bottleneck near the metal-insulator transition of the Hubbard model. An exact comparison is difficult, since their bottleneck arises in the midgap band of the dynamic mean-field theory doped Hubbard model, whereas in our quasiparticle-GW calculation this band arises due to nanoscale phase separation[30]. Nevertheless, they found that the bottleneck occurs well inside the pseudogap phase, where the near-FS band starts to become coherent. This is consistent with the transition in the Main text Fig. 13(b); note in particular that the bottleneck (red dot in the Main text Fig. 9) falls close to the coherent-incoherent transition (pink shaded region in Fig. 9 of the Main text). Ref. 31 also finds that strong bosonic fluctuations over a 'quasidegenerate distribution of $2\mathbf{p_F}$ ordering wave vectors' drives the pseudogap, although they find it to be predominantly in the charge sector, forming a 'resonant Peierls excitonic state'.

[1] Caprio, M. Cejnar, P. & Iachello, F. Excited state quantum phase transitions in many-body systems *Ann. Phys.* **323,** 1106 (2008).

[2] Pérez-Bernal, F. & Santos, L.F. Effects of excited state quantum phase transitions on system dynamics arXiv:1604.6851.

[3] Zhu, Y. *et al.* Tetragonal-orthorhombic structural modulation at low temperature in $La_{2-x}Ba_x$ CuO_4 *Phys. Rev. Lett.* **73,** 3026 (1994).

[4] Inoue, Y. Horibe, Y. & Koyama, Y. Evolution of low-temperature phases in a low-temperature structural transition of a La cuprate *Phys. Rev. B*56**,** 14176 (1997).

[5] Clogston, A.M. Upper limit for the critical field in hard superconductors *Phys. Rev. Lett.* **9,** 266 (1962).

[6] Chandrasekhar, B.S. A note on the maximum critical field of high-field superconductors *Appl. Phys. Lett.* **1,** 7 (1962).

[7] Collignon, C. *et a.* Fermi-surface transformation across the pseudogap critical point of the cuprate superconductor $La_{1.6-x}Nd_{0.4}Sr_xCuO_4$ *arXiv:1607.05693.*

[8] Fawcett, E. Spin-density-wave antiferromagnetism in chromium *Rev. Mod. Phys.* **60,** 209 (1988).

[9] Senthil, T. Vishwanath, A. Balents, L. Sachdev, S. & Fisher, M.P.A. Deconfined quantum critical points *Science* **303,** 1490-1494 (2004).

[10] Chen, K.-S. Meng, Z.Y. Pruschke, T. Moreno, J. & Jarrell, M. Lifshitz transition in the two-dimensional Hubbard model *Phys. Rev. B*86**,** 165136 (2012).

[11] Markiewicz, R.S. & Vaughn, M.T. Classification of the Van Hove scenario as an SO(8) spectrum-generating algebra. *Phys. Rev. B*57**,** 14052(RC) (1998).

[12] Zhang, S.-C. A unified theory based on SO(5) symmetry of superconductivity and antiferromagnetism *Science* **275,** 1089 (1997).

[13] Ivanchenko, Y.M., & Lisyansky, A.A. *Physics of Critical Fluctuations* (Springer, New York, 1995).

[14] McMillan, W.L. Microscopic model of charge-density waves in $2HTaSe_2$. *Phys. Rev. B*16**,** 643-650 (1977).

[15] Das, T. Markiewicz, R.S. & Bansil, A. Intermediate coupling model of the cuprates. *Advances in Physics* **63,** 151-266 (2014).

[16] Maier, T.A. Jarrell, M. Schulthess, T.C. Kent, P.R.C. & White, J.B. Systematic study of d-wave superconductivity in the 2D repulsive Hubbard model *Phys. Rev. Lett.* **95,** 237001 (2005).

[17] Fratino, L. Sémon, P. Sordi, G. & Tremblay, A.-M. S. Pseudogap and superconductivity in two-dimensional doped charge-transfer insulators *Phys. Rev. B*93**,** 245147 (2016).

[18] González, J. Guinea, F. & Vozmediano, M. A. H. Kinematics of electrons near a Van Hove singularity *Phys. Rev. Lett.* **84,** 4930 (2000).

[19] Toschi, A. Katanin, A.A. & Held, K. Dynamical vertex approximation: A step beyond dynamical mean-field theory *Phys. Rev. B*75**,** 045118 (2007).

[20] Katanin, A.A. Toschi, A. & Held, K. Comparing pertinent effects of antiferromagnetic fluctuations in the two- and three-dimensional Hubbard model *Phys. Rev. B*80**,** 075104 (2009).

[21] Toschi, A. Rohringer, G. Katanin, A.A. & Held, K. Ab initio calculations with the dynamical vertex approximation *Ann. Phys. (N.Y.)* **523,** 698-705 (2011).

[22] Rubtsov, A.N. Katsnelson, M.I. & Lichtenstein, A.I. Dual fermion approach to nonlocal correlations in the Hubbard model *Phys. Rev. B*77**,** 033101 (2008).

[23] Hafermann, H. *et al.* Efficient perturbation theory for quantum lattice models *Phys. Rev. Lett.* **102,** 206401 (2009).

[24] Rohringer, G. *et al.* One-particle irreducible functional approach: A route to diagrammatic extensions of the dynamical mean-field theory *Phys. Rev. B*88**,** 115112 (2013).

[25] Gunnarsson, O. *et al.* Fluctuation diagnostics of the electron self-energy: origin of the pseudogap physics *Phys. Rev. Lett.* **114,** 236402 (2015).

[26] Badoux, S. *et al.* Change of carrier density at the pseudogap critical point of a cuprate superconductor *Nature*, published online doi:10.1038/nature16983.

[27] Tam, K.-M. *et al.* Solving the parquet equations for the Hubbard model beyond weak coupling *Phys. Rev. E*87**,** 013311 (2013).

[28] Li, G. Wentzell, N. Pudleiner, P. Thunström, P. & Held, K. Efficient implementation of the parquet equations – role of the reducible vertex function and its kernel approximation *arXiv:1510.03330.*

[29] Sayyad, S. & Eckstein, M. Slowdown of the electronic relaxation close to the Mott transition *Phys. Rev. Lett.* **117,** 096403 (2016).

[30] Mistark, P. Markiewicz, R.S. & Bansil, A. Nanoscale phase separation in deeply underdoped $Bi_2Sr_2CuO_{6+\delta}$ and $Ca_2CuO_2Cl_2$ *Phys. Rev. B*91**,** 140501(R) (2015).

[31] Kloss, T. Montiel, X. & Pépin, C. Resonant Peierls excitonic state as a new mechanism for the pseudogap and T-linear resistivity in cuprate superconductors *arXiv:1510.3038.*
